# Frozen elephant trunk versus conventional proximal repair of acute aortic dissection type I

**DOI:** 10.3389/fcvm.2024.1326124

**Published:** 2024-03-15

**Authors:** Nora Göbel, Simone Holder, Franziska Hüther, Yasemin Anguelov, Dorothee Bail, Ulrich Franke

**Affiliations:** Department of Cardiovascular Surgery, Robert-Bosch-Hospital, Stuttgart, Germany

**Keywords:** frozen elephant trunk, aortic dissection type I, aortic arch surgery, long-term outcomes, mortality, neurologic deficit, reintervention

## Abstract

**Objective:**

The extent of surgery and the role of the frozen elephant trunk (FET) for surgical repair of acute aortic dissection type I are still subjects of debate. The aim of the study is to evaluate the short- and long-term results of acute surgical repair of aortic dissection type I using the FET compared to standard proximal aortic repair.

**Methods:**

Between October 2009 and December 2016, 172 patients underwent emergent surgery for acute type I aortic dissection at our center. Of these, *n* = 72 received a FET procedure, while the other 100 patients received a conventional proximal aortic repair. Results were compared between the two surgery groups. The primary endpoints included 30-day rates of mortality and neurologic deficit and follow-up rates of mortality and aortic-related reintervention.

**Results:**

Demographic data were comparable between the groups, except for a higher proportion of men in the FET group (76.4% vs. 60.0%, *p* = 0.03). The median age was 62 years [IQR (20), *p* = 0.17], and the median log EuroSCORE was 38.6% [IQR (31.4), *p* = 0.21]. The mean follow-up time was 68.3 ± 33.8 months. Neither early (FET group 15.3% vs. proximal group 23.0%, *p* = 0.25) nor late (FET group 26.2% vs. proximal group 23.0%, *p* = 0.69) mortality showed significant differences between the groups. There were fewer strokes in the FET patients (FET group 2.8% vs. proximal group 11.0%, *p* = 0.04), and the rates of spinal cord injury were similar between the groups (FET group 4.2% vs. proximal group 2.0%, *p* = 0.41). Aortic-related reintervention rates did not differ between the groups (FET group 12.1% vs. proximal group 9.8%, *p* = 0.77).

**Conclusion:**

Emergent FET repair for acute aortic dissection type I is safe and feasible when performed by experienced surgeons. The benefits of the FET procedure in the long term remain unclear. Prolonged follow-up data are needed.

## 1 Introduction

The frozen elephant trunk (FET) technique has evolved into an effective and established therapy to treat the aortic arch and proximal descending aorta in a single procedure (1). In degenerative aortic aneurysm surgery, the second-stage downstream therapy, either open or endovascular, was substantially facilitated. However, the benefits of FET implantation in the setting of acute aortic dissection are still not fully elucidated. Therefore, the role of FET in acute dissection remains a topic of controversial discussion (2). In DeBakey type I dissection, conventional proximal aortic repair usually results in residual false lumen patency in the downstream aorta, which has been shown to be associated with elevated risk of dilation, rupture, and mortality (3). The FET enables stabilization of the distal arch and proximal descending aorta and therefore promotes aortic remodeling effectively (4). Especially in patients with distal re-entries and/or malperfusion, the FET enables false lumen decompression and restoration of true lumen perfusion (5). However, FET surgery is far more complex, potentially elevating perioperative risk in an already high-risk acute situation. Obviously, long-term benefits of the FET can only apply when the patient survives the acute operation. Moreover, not every attending surgeon is similarly skilled and familiar with this complex technique and inherited pitfalls. It has been proved that FET implantation in acute dissection is safe in experienced hands (6). We therefore compared our results of the FET vs. conventional proximal arch repair in DeBakey type I acute aortic dissections in a real-world setting.

## 2 Methods

### 2.1 Patients

Interrogation of our prospectively collected dissection database revealed 172 consecutive patients with DeBakey type I acute aortic dissection who underwent emergent surgery at our center between October 2009 and December 2016. Of these, 72 patients were treated using the FET technique (FET group), whereas 100 patients received conventional proximal surgical aortic repair (proximal group). Outcomes were retrospectively compared according to the type of surgery performed.

The study was approved by the authorized ethics review committee (University of Tuebingen, No. 069/2019BO2). Informed consent was obtained from all patients.

### 2.2 Endpoints

Primary endpoints included 30-day rates of mortality and neurologic deficit and follow-up rates of mortality and aortic-related reintervention including reoperation. Secondary endpoints included operative times, length of hospital and ICU stay, acute kidney injury, type of reintervention (aortic root vs. distal aorta), and mean time to reintervention.

The neurologic deficit was defined as stroke or spinal cord injury as assessed by imaging and/or specialist neurologic clinical examination. Acute kidney injury was defined according to KDOQI stage 3, i.e., requiring renal replacement therapy. Aortic-related reintervention was defined as any surgery on the aortic root and/or surgery or endovascular therapy on the distal aorta due to progressive aortic dilation or rupture.

### 2.3 Operative details

The institutional standard for the surgery of acute aortic dissection is an all-comers approach without delay in the timing of surgery, which is performed by the attending surgeon. In short, whenever possible, the right axillary artery is our primary arterial cannulation site, and we prefer direct vessel cannulation. Femoral or direct aortic arterial cannulation is rarely performed. Cardiocirculatory arrest is induced under moderate hypothermia of 28°C, combined with bilateral antegrade cerebral perfusion. The distal anastomosis is always accomplished in an open manner under circulatory arrest to control and correct the aortic arch for secondary (re-)entries, if applicable. Every attending surgeon performs acute dissection surgery, but only a few are trained and skilled with the FET technique. If surgical expertise was available, indication for the FET implantation included entry/re-entry in the aortic arch, proximal descending aorta, distal malperfusion, young patient age, and appropriate size of the proximal descending aorta. FET was implanted in arch zone 3, according to the classification of Ishimaru (7). The supra-aortic vessels were reimplanted as islands. The E-vita Open Plus prosthesis (Jotec GmbH/Artivion, Hechingen, Germany) was used in all cases for the FET procedure. The stentgraft length was 15 cm, but only with the most recent implantations, we introduced the shorter 13 cm version. Dacron vascular prostheses (Hemashield, Getinge, Gothenburg, Sweden) were used for all other aortic replacements. Anastomoses were sutured using 3-0 or 4-0 polypropylene; the use of reinforcing felt strips varied according to the surgeons' preferences.

### 2.4 Follow-up

All patients underwent regular clinical and imaging follow-up on an ambulatory basis, with the first visit scheduled 3–6 months after surgery, followed by annual visits thereafter. In stable conditions, the interval between visits was extended to every 2 years.

### 2.5 Statistical analysis

All statistical calculations were performed using SPSS Version 26.0 (IBM SPSS Statistics for Windows, IBM Corp., Armonk, New York, United States). Categorical data are reported as numbers and percentages, and continuous data are reported as means ± standard deviations or medians with interquartile ranges, as appropriate. The assumption of normal distribution was tested with the Kolmogorov–Smirnov test. A comparison of categorical data was performed with Fisher's exact test or chi-square test. Normally distributed continuous data were compared using the *t*-test, and while the Mann–Whitney *U*-test was applied if the normal distribution was not met. Kaplan–Meier estimates were used to analyze the rates of survival and reintervention during follow-up, with groups compared using the log-rank test. Values of *p* < 0.05 were considered statistically significant.

## 3 Results

Demographic data were comparable between the groups, except for a higher proportion of men in the FET group (76.4% vs. 60.0%, *p* = 0.03). The median age was 62 years [IQR (20), *p* = 0.17], and the median log EuroSCORE was 38.6% [IQR (31.4), *p* = 0.21]. In addition, groups were well balanced regarding the mode of preoperative presentation: overall, 6.4% had previous cardiac surgery (*p* = 0.76), 22.1% presented in cardiogenic shock (*p* = 0.85), and 29.7% already had a preoperative neurologic deficit (*p* = 0.43). Complete demographics are summarized in Table 1.

**Table 1:** Demographic data.

Variable	All (*n* = 172)	FET group (*n* = 72)	Proximal group (*n* = 100)	*p*-Value
Age (y)	62 [20.0]	60 [17.0]	64 [21.8]	0.17
Male sex	66.9%	76.4%	60.0%	0.03
BMI (kg/qm)	26.0 [6.0]	26.5 [6.1]	26.0 [5.0]	0.63
Log ES (%)	38.6 [31.4]	36.3 [30.6]	42.1 [34.9]	0.21
Hypertension	89.5%	94.4%	86.0%	0.08
Diabetes	6.4%	4.2%	8.0%	0.36
COPD	10.5%	13.9%	8.0%	0.31
Chronic kidney disease	26.7%	22.9%	33.0%	0.22
Previous cardiac surgery	6.4%	5.6%	7.0%	0.76
Preop. CS	22.1%	20.8%	23.0%	0.85
Tamponade	23.8%	13.9%	31.0%	0.01
Preop. CPR	7.0%	2.8%	10.1%	0.08
Preop. ventilation	15.1%	9.7%	19.0%	0.13
Preop. neurologic deficit	29.7%	26.4%	32.0%	0.43
Preop. malperfusion	30.8%	26.4%	34.0%	0.32

FET, frozen elephant trunk; y, years; BMI, body mass index; kg/qm, kilograms per square meter; ES, EuroSCORE; %, percent; COPD, chronic obstructive pulmonary disease; CS, cardiogenic shock; CPR, cardiopulmonary resuscitation. Categorical variables are presented as percentages, and continuous variables are presented as medians with [interquartile ranges].

Intraoperative procedural times were all significantly longer in the FET group: operative time 355 ± 79 vs. 312 ± 87 min (*p* < 0.001), cardiopulmonary bypass time 237 ± 62 vs. 209 ± 68 min (*p* = 0.001), cross-clamp time 160 ± 36 vs. 132 ± 42 min (*p* < 0.001), and circulatory arrest time 72 ± 16 vs. 38 ± 25 min (*p* < 0.001). Application of antegrade cerebral perfusion was 100% in the FET group and 93% in the proximal group, which proved statistically significant (*p* = 0.02). There were significantly more aortic root repairs in the FET group (48.6% vs. 32.0%, *p* < 0.001). However, concomitant bypass surgeries (8.3% vs. 10.0%, *p* = 0.80) were distributed similarly between the groups; see Table 2 for intraoperative details.

**Table 2:** Intraoperative details.

Variable	All (*n* = 172)	FET group (*n* = 72)	Proximal group (*n* = 100)	*p*-Value
Operative time (min)	330 ± 85	355 ± 79	312 ± 87	<0.001
CPB time (min)	221 ± 67	237 ± 62	209 ± 68	0.001
Cross-clamp time (min)	144 ± 42	160 ± 36	132 ± 42	<0.001
Circulatory arrest time (min)	53 ± 27	72 ± 16	38 ± 25	<0.001
Axillary arterial cannulation	82.6%	87.5%	79.0%	0.16
Antegrade cerebral perfusion	95.3%	100%	93.0%	0.02
Aortic root replacement	37.8%	27.8%	45.0%	0.02
Aortic root repair	38.9%	48.6%	32.0%	<0.001
CABG	9.3%	8.3%	10.0%	0.80

FET, frozen elephant trunk; min, minutes; CPB, cardiopulmonary bypass; CABG, coronary artery bypass grafting. Categorical variables are presented as percentages, and continuous variables are presented as means ±standard deviations.

The 30-day mortality rate was 15.3% in the FET group, which was lower than the 23.0% in the proximal group, but the difference did not reach statistical significance (*p* = 0.25). Overall postoperative neurologic deficit did not differ between the groups (14.5%, *p* = 0.13). There were significantly fewer strokes in the FET patients (2.8% vs. 11.0%, *p* = 0.04), and the rates of spinal cord injury were similar between the groups (FET group 4.2% vs. proximal group 2.0%, *p* = 0.41).

**Figure 1:**
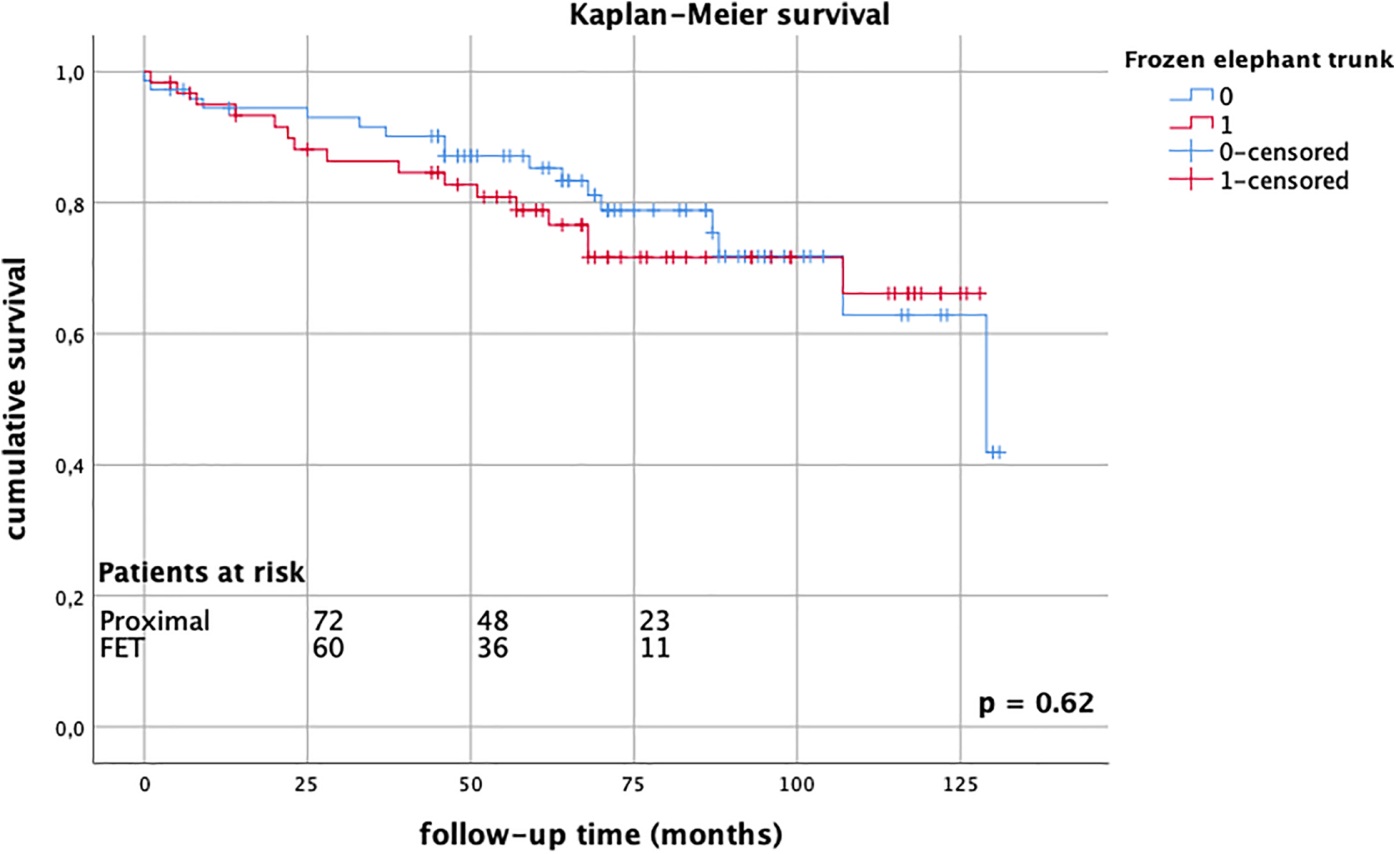
Kaplan–Meier survival estimates comparing FET group (red) vs. proximal group (blue) up to 10 years, without significant differences between groups, log-rank test *p* = 0.62.

**Figure 2:**
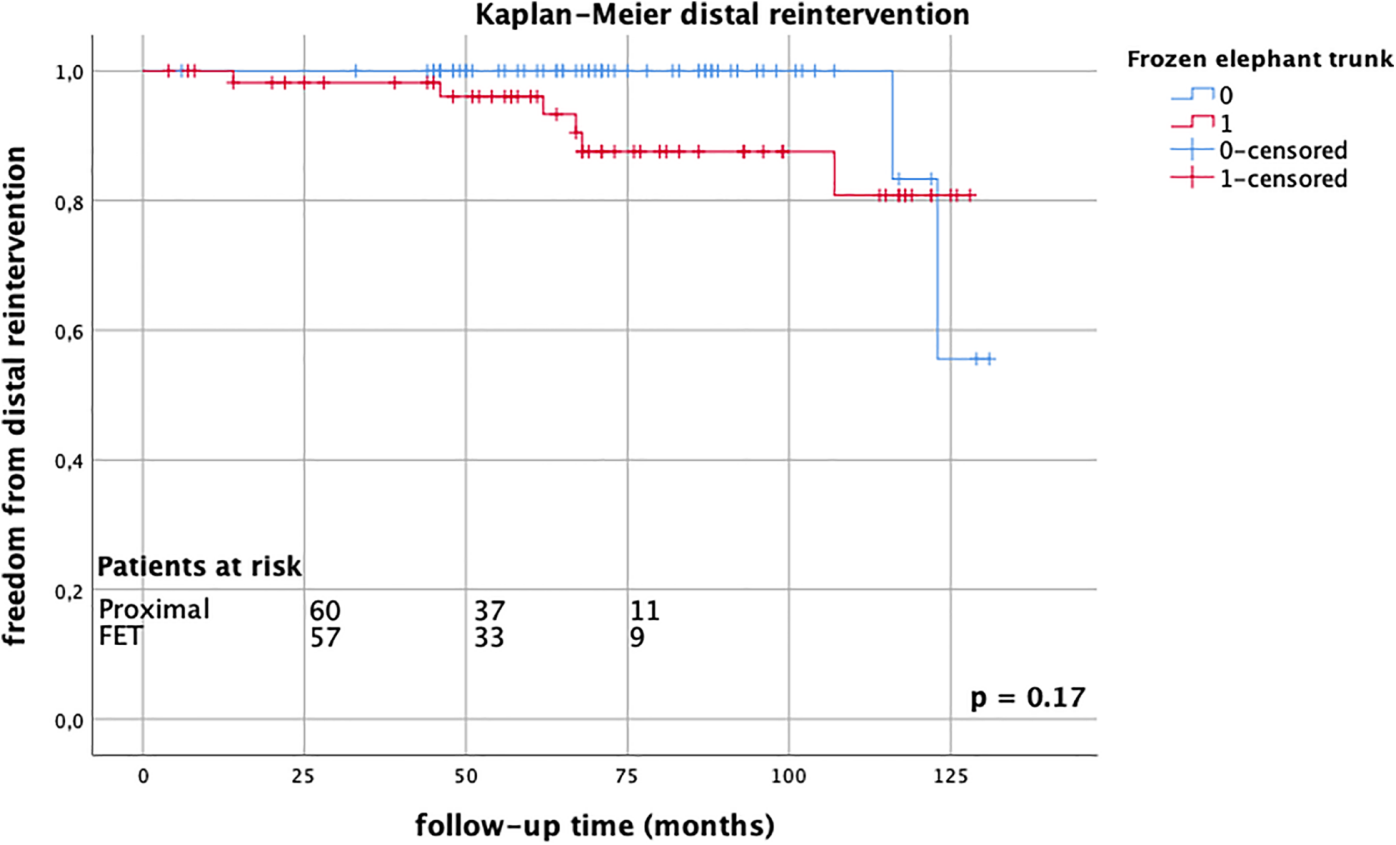
Kaplan–Meier estimates for distal reintervention comparing FET group (red) vs. proximal group (blue) up to 10 years, without significant differences between groups, log-rank test *p* = 0.17.

Follow-up was completed in 98.8% of cases. The mean follow-up time was 68.3 ± 33.8 months (range 1–131 months). Late mortality rates were well comparable between the groups (FET group 26.2% vs. proximal group 23.0%, *p* = 0.69), Figure 1. There were 13 reinterventions in follow-up, with 7 occurring in the FET group and 6 occurring in the proximal arch group; one patient required combined proximal and distal aortic repair. Overall aortic-related reintervention rates did not differ between the groups (FET group 12.1% vs. proximal group 9.8%, *p* = 0.14), nor did distal reintervention rates alone (FET group 8.3% vs. proximal group 3.0%, *p* = 0.12), Figure 2. The mean time to reintervention was 40.6 ± 34.9 (*p* = 0.91). All outcome data are summarized in Table 3.

**Table 3:** Outcomes.

Variable	All (*n* = 172)	FET group (*n* = 72)	Proximal group (*n* = 100)	*p*-Value
Acute kidney injury	18.0%	23.6%	14.4%	0.16
Postop. new neurologic deficit	14.5%	9.7%	18.0%	0.13
Temporary	4.1%	2.8%	5.0%	0.47
Stroke	7.6%	2.8%	11.0%	0.04
Spinal cord injury	2.9%	4.2%	2.0%	0.41
Length of ICU stay (d)	5 [6.0]	5 [6.0]	5 [8.0]	0.60
Length of hospital stay (d)	19 [19.0]	20 [22.0]	18 [19.8]	0.46
30-day mortality (%)	19.8%	15.3%	23.0%	0.25
FU reintervention	7.6%	12.1%	9.8%	0.14
Proximal	2.9%	1.4%	4.0%	0.77
Distal	5.2%	8.3%	3.0%	0.12
Time to reintervention (m)	40.6 ± 34.9	41.7 ± 35.6	39.3 ± 37.4	0.91
FU mortality	24.4%	26.2%	23.0%	0.69

FET, frozen elephant trunk; ICU, intensive care unit; FU, follow-up; d, days; m, months. Categorical variables are presented as percentages, and continuous variables presented given as medians with [interquartile ranges] or means ±standard deviations.

## 4 Discussion

The FET technique is increasingly used in acute aortic dissection surgery, but its role remains unclear. Our data confirm the safety and feasibility of FET implantation in acute aortic dissection but question the propagated beneficial long-term effects. At a mean follow-up time of 5.7 years, we did not observe any significant differences in survival and reintervention rates between the FET group and proximal repair-only patients.

Our 30-day mortality rates were comparable between the groups, regardless of the extent of surgery, as were the rates of postoperative new neurologic deficits. Moreover, these results are well comparable to contemporary reported outcomes: Overall 30-day mortality ranges between 16.9% in the GERAADA registry and 19.7% in the IRAD to 22% in the French registry vs. 19.2% at our center (8–12). Rates of postoperative permanent neurologic deficit range between 9.5% and 18.7% and were 10.5% in our patients (10, 11, 13–16).

Due to dismal outcomes, we discontinued FET implantation in patients after resuscitation during the study period; however, these patients represent only a small fraction of the cohort, but may explain the slightly elevated mortality rates in the proximal repair group due to selection bias, although it did not reach statistical significance. Uehara et al. proved the unfavorable prognosis of these patients and even suggested withdrawal of surgery on patients with prolonged cardiopulmonary resuscitation or without return of circulation after pericardiotomy due to the reduced chances of surviving without any neurologic deficit (17).

Of note, some proximal arch repair patients also experienced spinal cord injuries, without statistically significant differences between groups. Similar findings have been published by Poon et al. based on data from the large international ARCH registry: using propensity-score matching FET and conventional arch replacement, patients did not exhibit differences in stroke or spinal cord injury rates, emphasizing the multifactorial etiology of this devastating complication (16).

With both techniques parallelly in use, we provide a valid and appropriate comparison cohort, as all the perioperative settings and management are identical, and no unknown confounders need to be considered. Direct comparative studies reporting long-term outcomes are hardly found in the literature. Yoshitake et al. reported superior long-term survival rates with the FET technique, but reintervention rates were similar between FET and non-FET patients (18). These findings were confirmed by a recent meta-analysis comparing long-term Kaplan–Meier derived data of total arch and proximal arch repair patients: overall survival was better in the total arch group, but the risk of reoperation did not differ significantly. However, this held only true for the first 7 years after the index operation; thereafter, total arch patients had lower reoperation rates (19).

As early as 2015, Shrestha et al. already verbalized the concerns of overtaxing FET implantations in acute dissections (“are we pushing the limits too far”), emphasizing that indications have to be stated well-considered (20). A more restrictive approach seems to be reasonable in most hands and situations. A contemporary international multicenter study confirms the safety of a more limited surgical approach, showing even inferior survival rates with total arch repair and similar reoperation rates in follow-up compared to proximal arch surgery (9). Concentrating FET surgery in acute type A dissection repair to only experienced aortic centers might be a reasonable consequence (21, 22).

Neurologic complications are the most feared in dissection surgery as they seriously impact the quality of life and survival of these patients. With the FET technique, we face a dilemma: the longer the stentgraft portion with deep coverage of the descending aorta, the better the rate of aortic remodeling; however, at the same time, the risk of spinal cord injury is elevated (23). Shortening the stent-graft length and moving the proximal implantation level to arch zone 2 have nearly eliminated the devastating complication of spinal cord injury but carry the elevated risk of false lumen patency with the increased need for secondary interventions (24). Stroke rates were favorably low in our FET patients (2.8%), despite significantly longer circulatory arrest times than proximal arch repairs, which may be attributable to a strict cerebral perfusion protocol, whereas 7% of our conventional proximal group did not receive selective brain perfusion. Contemporary data report stroke rates of 2.7%–18% (21).

So far, secondary interventions have been associated with a substantial risk of mortality (14%–40%) (25, 26). The FET technique provides an excellent landing zone for further interventions, enabling endovascular therapy in most patients. In contrast, after proximal arch repair, a high percentage of patients required open surgery for distal reoperation, carrying a relevant risk of mortality (18). In the future, with further development of interventional and hybrid procedures, the second-stage intervention becomes potentially safer. Some contemporary data already demonstrate excellent outcomes of redo surgeries after limited dissection type I repair, as they can safely be performed in an elective setting at experienced centers (27, 28).

In our study, the follow-up rates of reintervention did not differ between the groups. Potentially, we are facing a paradox: while the FET easily enables distal endovascular extension, this procedure could be performed more liberally (and safer) than open surgery, which is more often needed after hemiarch repair (18); these patients may be deemed unsuitable for endovascular and inoperable for open reoperation. Moreover, FET patients adhere more strictly to follow-up visits. Therefore, more distal aortic dilations could be diagnosed in these patients compared to proximal-only patients without long-term surveillance. Accordingly, An et al. reported the paradox, wherein patients with guideline-adherent postoperative surveillance exhibit higher rates of reinterventions and mortality than those without (29). Therefore, each reintervention should be indicated wisely.

Surprisingly, the long-term risk of mortality was not affected by the extent of index surgery in this study. This is in contrast to the results of Yoshitake et al., who reported a survival benefit for FET patients (18). The explanation lies beyond follow-up time: the mean follow-up time was 68.3 months in our study but only 46.0 months in the study by Yoshitake et al. However, our patient cohort might have been too small to find significant differences. Moreover, geographical differences between Asia and Europe may play a role (30). Nevertheless, the enthusiasm about FET implantation in acute dissection has been tarnished. Further data are needed to clarify the role of the FET in acute dissection surgery.

In conclusion, the application of the FET in acute aortic dissection is as safe and feasible as conventional proximal arch repair in our study. After a mean follow-up of 5.7 years, the rates of survival and reintervention were similar between the groups, irrespective of the initial extent of surgery. A limited approach may be reasonable according to the estimated life expectancy of patients.

### 4.1 Limitations

Limitations of the study include its retrospective nature and the relatively small size of the single-center patient cohort.

## Data Availability

The raw data supporting the conclusions of this article will be made available by the authors without undue reservation.
